# Awakening

**Published:** 1889-09-07

**Authors:** 


### Words of Consolation.

### AWAKENING.

*(For Reading to the Sick.)*

Let us picture to ourselves a ship in a far-off sea, tossed by stormy waves and almost lost. The mariners are at their wits' end and are calling on their gods to save them, while one passenger lies calmly sleeping in the sides of the ship. The shipmaster is astonished at the sight and asks, "What meanest thou, O sleeper? Arise, call upon thy God, if so be that God will think upon us that we perish not." The man was Jonah, fleeing from the wrath of God. We are reminded also of another ship, many years after, on the Sea of Gennesrrat, in which was another sleeper, even our Lord Jesus Christ—a greater than Jonah was there. Round that vessel, too, winds and waves were beating in their fury, so that it was about to sink. The disciples were afraid, and besought Him to arise, crying, "Help, Lord, or we perish!" Then He arose, and rebuked the winds and the sea, and there was a great calm!

The whole Church is a ship, with passengers of different sorts, tossed by the waves of this troublesome world. Those who are on board, in their fear of the storm and the agitation of the sea, flee to Christ to awaken Him who is apparently slumbering. But it is we who are sleeping in sin and who must arouse, for "He watching over Israel neither slumbers nor sleeps." Each one must say to himself "Awake thou that sleepest, and arise from the dead, and Christ shall give thee life." Strange to tell a dead man to arise, you think! No, the sinner is not dead, only lying in a death like state of trespasses and sins. Sleep is indeed a very fit emblem of the state of a careless, thoughtless sinner, for though the body is full of life, it is incapable of performing its accustomed actions. God demands obedience. He calls for love, but the sleeper is weak, unable, and, what is more, disinclined for exertion. Sleep often brings to the careless man pleasant dreams of safety while he is drifting on to destruction; he is deaf to the calls of duty, and enjoys the satisfaction which his isions of worldly success bring before him. To such a one, fast bound by the slumber of sin, we may well say, What dost thou mean by such indifference? Rise up while there is time, and call upon the Lord thy God to help, lest thou perish. Why dost thou hesitate in a matter of life or death? If thou art not in earnest, God is. Meanest thou to sin against so much goodness? Arise, call upon thy God, arise from the miserable state of sloth in which thou hast been living and pray. Prayer is one of the first signs of life, and the gasps and sobs of returning animation will be turned into prayers by the Holy Spirit, which helpeth our infirmities. Call, then, O careless one, call upon thy God, and He will hear and save thee as a waif from the bottomless ocean, as a brand from the devouring fire. Thou hast required many a buffet, many a wave of sorrow and suffering to rouse thee, sunk asleep in the pleasures or cares of life, but now thou hast a time given thee for recollection; and though thou art at first dizzy and scared as the light breaks on thine unaccustomed eyes, use this time of rest to repent of thy sins and make good resolutions for the future, which by God's help thou shalt surely carry out. The penitent shall not perish, his sins shall be blotted out as a cloud, yea, as a thick cloud; God will renew in him his faith and love, which were decayed and ready to perish, and will admit him afresh, like the prodigal son, into His house and family, and bless him with the hope of everlasting life.

And when death comes, victory and peace shall be his, and an abundant entering into the kingdom of our Lord Jesus Christ, where there shall be no more death, neither sorrow, nor crying, for the former things are passed away.

"Save, Lord, we perish!" was their cry,
"O, save us in our agony!"
Thy word above the storm rose high,
"Peace be still."

So, when our life is clouded o'er,
And storm-winds drift us from the shore,
Say, lest we sink to rise no more,
"Peace be still."

